# In Silico Screening and In Vitro Activity Measurement of Javamide Analogues as Potential p38 MAPK Inhibitors

**DOI:** 10.3390/ijms18122704

**Published:** 2017-12-13

**Authors:** Jae B. Park

**Affiliations:** Diet, Genomics, and Immunology Laboratory, Bldg. 307C, Rm. 131, BHNRC, ARS, USDA, Beltsville, MD 20705, USA; jae.park@ars.usda.gov; Tel.: +1-301-504-8365; Fax: +1-301-504-9062

**Keywords:** in silico screening, javamide analogues, p38 MAPK inhibitors

## Abstract

p38 Mitogen-activated protein kinase (p38 MAPK) is a protein kinase critically involved in the progress of inflammation/stress-associated diseases. Our data suggested that javamide analogues may contain strong anti-inflammation activities, but there is little information about their effects on p38 MAPK. Therefore, in this paper, the effects of thirty javamide analogues on p38 MAPK were investigated using in silico screening and in vitro p38 MAPK assay methods. The javamide analogues were synthesized and their chemical structures were confirmed using nuclear magnetic resonance (NMR) spectroscopic methods. Then, the javamide analogues were screened using an in silico modeling program. The screened analogues demonstrated a wide range of binding energy (ΔE; −20 to −39) and several analogues with ΔE; −34 to −39 showed strong binding affinity to p38 MAPK. In vitro p38 MAPK assay, the kinase was significantly inhibited by the analogues with great binding energy (ΔE; −34 to −39) and in silico scores (Avg. score; −27.5 to −29.3). Furthermore, the comparative analysis of both assays showed a positive correlation between the in silico scores and p38 MAPK inhibition. In fact, the javamide analogues with top five in silico scores (Avg. score; −27.5 to −29.3) were found to inhibit p38 MAPK by 27–31% (*p* < 0.05) better than those with less scores (ΔE < −27.0). Especially, javamide-II-*O*-ethyl ester with relatively high in silico score (Avg. score; −29.2) inhibited p38 MAPK (IC_50_ = 9.9 μM) a little better than its methyl ester with best in silico score (Avg. score; −29.3). To support the ability to inhibit p38 MAPK, the treatment of javamide-II-ethyl and -methyl esters could suppress the production of IL-8 and MCP-1 protein significantly by 22–73% (*p* < 0.05) in the differentiated THP-1 cells, and the inhibition was slightly stronger by the ethyl ester than the methyl ester. Altogether, this study suggests that javamide-II-*O*-ethyl ester may be a most potent p38 MAPK inhibitor among the tested compounds and the combining in silico and in vitro assay approach may be a useful and efficient solution as a functional screening approach in searching new lead compounds for targeted molecules.

## 1. Introduction

In silico screening (a computer-aided virtual screening) is one of useful and alternative approaches complementing/substituting conventional high throughput screening which is often expensive and time-consuming. Because in silico screening can identify new inhibitors for targeted molecules quickly and effectively, it has emerged as a more suitable screening method in finding candidate compounds than traditional high throughput screening. In fact, in silico screening method has been successfully utilized in finding biologically active compounds against targeted molecules such as Sirt, histone deacetylase, protein kinases, and viral proteases [1,2,3,4,5]. In utilization, in silico screening can be performed in two different way; a ligand-based design (LBD) or structure-based design (SBD) approach. The former uses criteria from chemicals properties of known active ligands, meanwhile the latter utilizes criteria from the 3D structure properties of targeted proteins [5,6,7,8,9,10]. Therefore, the SBD approach is preferable to the LBD approach when the molecular structure of a targeted protein is available. In the SBD approach, two standard operations are performed; “docking” and “scoring”. Typically, the first is performed by docking a collection of candidate compounds into active sites of a targeted protein and determining their optimal orientations by conformational, translational, and rotational movement in the active sites. Subsequent scoring derived from the docking is utilized to assess the correct binding mode of the complexes with candidate compounds, enabling to rank candidate compounds by binding energy (ΔE), which allows identification and prioritization of lead compounds [9,10].

p38 MAPK is a mitogen-activated protein kinase responsive to inflammation/stress stimuli such as cytokines, stress, ultraviolet irradiation, heat shock, and osmotic shock. There are four isoforms of p38 MAPK, p38 α (MAPK14), -β (MAPK11), -γ (MAPK12 or ERK6) and -Δ (MAPK13 or SAPK4). Among them, p38 α is a relatively well-studied kinase deeply involved in the biosynthesis of several inflammatory cytokines such as TNF-α and IL-1β. Because p38 α is profoundly involved in multiple signaling processes activated during inflammation, this kinase is considered as a key target molecule for regulating inflammatory cytokine production. For this reason, p38 inhibitors have been sought after for years as potential therapeutic agents for treating inflammatory diseases [11,12,13]. In fact, some p38 inhibitors have been demonstrated to inhibit the production of several inflammatory cytokines/chemokines such as interleukin (IL)-1, IL-8, TNF-α and MCP-1, thereby providing protective effects on chronic inflammatory diseases including arthrosclerosis and cardiovascular disease [11,12,13,14,15]. However, the majority of p38 inhibitors have not been successful due to high toxicity and poor selectivity [15]. Therefore, there is still on-going effort for finding p38 inhibitors with less side effects and great selectivity [14,15]. Interestingly, our data suggest that one of javamide-II analogues (javamide-II-*O*-methyl ester) can inhibit p38 MAPK better than javamide-II in the cells [16], suggesting the likelihood that the ester forms may inhibit p38 MAPK stronger than the parent compounds. Therefore, in this paper, javamide analogues were synthesized and their potential effects on p38 MAPK inhibition were investigated using in silico modeling, in vitro p38 MAPK assay and a cell culture model.

## 2. Results

### 2.1. Chemical Synthesis and NMR Analysis of Javamide Analogues

**Figure 1:**
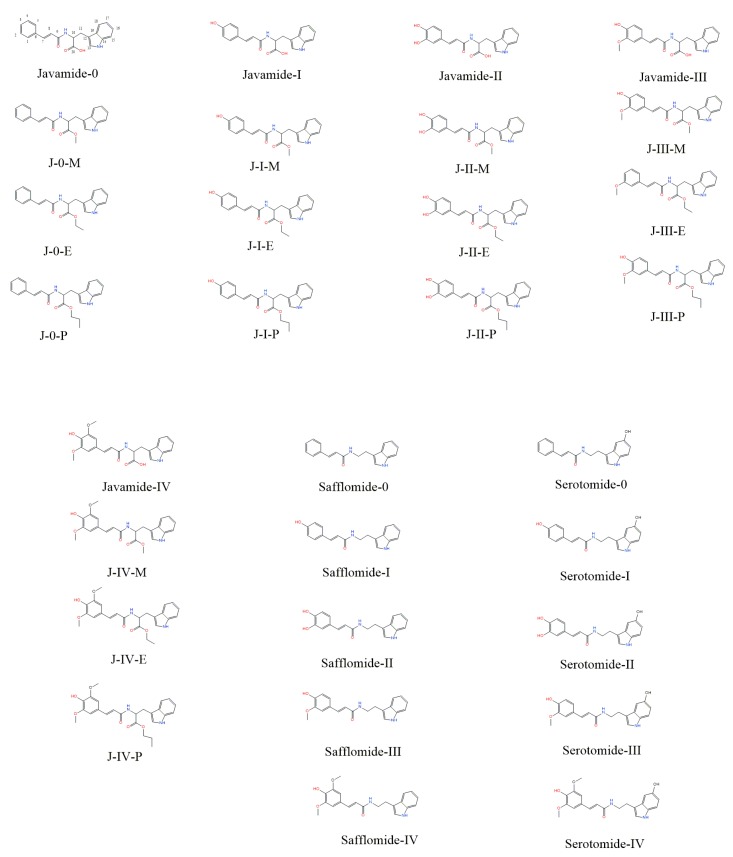
Chemical structures of javamide analogues. The chemical synthesis was performed as described in “Materials and Methods”. M, E and P represent methyl, ethyl and propanyl, respectively. The chemical structures were verified using nuclear magnetic resonance (NMR) spectroscopic method.

Javamide analogues (Figure 1) were synthesized as described in “Materials and Methods”. The final products were purified by high-performance liquid chromatography (HPLC) (Waters, Milford, MA, USA) and analyzed by Nuclear magnetic resonance (NMR) as described previously [16,17,18].

**Javamide-0.** NMR data: ^1^H-NMR (*d*_6_-DMSO, 400 MHz) δ 7.58 (1H, d, *J =* 8.2 Hz, H-18), 7.62 (1H, d, *J =* 8.2 Hz, H-1/H-5), 7.38 (1H, t, *J =* 7.3 Hz, H-2/4), 7.37 (1H, d, *J =* 15.6 Hz, H-7), 7.33 (1H, d, *J =* 7.8 Hz, H-15), 7.20 (1H, s, H-13), 7.06 (1H, t, *J =* 7.3 Hz, H-16), 6.98 (1H, t, *J =* 7.3 Hz, H-17), 6.46 (1H, d, *J =* 15.6 Hz, H-8), 4.72 (1H, t, *J =* 7.3 Hz, H-10), 3.24 (1H, dt, *J =* 6.0, 6.9 Hz, H-11), 12.89 (1H, br s, OH-a′), 10.79 (1H, br s, NH-β), 8.38 (1H, br s, NH-α); ^13^C-NMR (*d*_6_-DMSO, 100 MHz) 174.7 (C, C-20), 166.8 (C, C-9), 141.7 (C, C-7), 136.4 (C, C-14), 128.5 (C, C-1/5), 135.2 (C, C-6), 128.6 (C, C-2/4), 127.9 (C, C-3), 127.4 (C, C-19), 124.0 (C, C-8), 123.0 (C, C-13), 121.7 (C, C-16), 119.8 (C, C-17), 118.8 (C, C-18), 111.5 (C, C-15), 109.3 (C, C-12), 60.3 (C, C-10), 28.2 (C, C-11).

**Javamide-0-methyl**. NMR data: ^1^H-NMR (*d*_6_-DMSO, 400 MHz) δ 7.58 (1H, d, *J =* 8.2 Hz, H-18), 7.62 (1H, d, *J =* 8.2 Hz, H-1/H-5), 7.38 (1H, t, *J =* 7.3 Hz, H-2/4), 7.37 (1H, d, *J =* 15.6 Hz, H-7), 7.33 (1H, d, *J =* 7.8 Hz, H-15), 7.20 (1H, s, H-13), 7.06 (1H, t, *J =* 7.3 Hz, H-16), 6.98 (1H, t, *J =* 7.3 Hz, H-17), 6.46 (1H, d, *J =* 15.6 Hz, H-8), 4.72 (1H, t, *J =* 7.3 Hz, H-10), 3.24 (1H, dt, *J =* 6.0, 6.9 Hz, H-11), 12.89 (1H, br s, OH-a′), 10.79 (1H, br s, NH-β), 8.38 (1H, br s, NH-α), 3.66 (1H, s, CH_3_-1); ^13^C-NMR (*d*_6_-DMSO, 100 MHz) 174.7 (C, C-20), 166.8 (C, C-9), 141.7 (C, C-7), 136.4 (C, C-14), 128.5 (C, C-1/5), 135.2 (C, C-6), 128.6 (C, C-2/4), 127.9 (C, C-3), 127.4 (C, C-19), 124.0 (C, C-8), 123.0 (C, C-13), 121.7 (C, C-16), 119.8 (C, C-17), 118.8 (C, C-18), 111.5 (C, C-15), 109.3 (C, C-12), 60.3 (C, C-10), 28.2 (C, C-11), 51.9 (C, C-CH_3_-1).

**Javamide-0-ethyl**. NMR data: ^1^H-NMR (*d*_6_-DMSO, 400 MHz) δ 7.58 (1H, d, *J =* 8.2 Hz, H-18), 7.62 (1H, d, *J =* 8.2 Hz, H-1/H-5), 7.38 (1H, t, *J =* 7.3 Hz, H-2/4), 7.37 (1H, d, *J =* 15.6 Hz, H-7), 7.33 (1H, d, *J =* 7.8 Hz, H-15), 7.20 (1H, s, H-13), 7.06 (1H, t, *J =* 7.3 Hz, H-16), 6.98 (1H, t, *J =* 7.3 Hz, H-17), 6.46 (1H, d, *J =* 15.6 Hz, H-8), 4.72 (1H, t, *J =* 7.3 Hz, H-10), 3.24 (1H, dt, *J =* 6.0, 6.9 Hz, H-11), 12.89 (1H, br s, OH-a′), 10.79 (1H, br s, NH-β), 8.38 (1H, br s, NH-α), 4.07 (1H, s, CH_3_-1), 1.21 (1H, s, CH_3_-2); ^13^C-NMR (*d*_6_-DMSO, 100 MHz) 174.7 (C, C-20), 166.8 (C, C-9), 141.7 (C, C-7), 136.4 (C, C-14), 128.5 (C, C-1/5), 135.2 (C, C-6), 128.6 (C, C-2/4), 127.9 (C, C-3), 127.4 (C, C-19), 124.0 (C, C-8), 123.0 (C, C-13), 121.7 (C, C-16), 119.8 (C, C-17), 118.8 (C, C-18), 111.5 (C, C-15), 109.3 (C, C-12), 60.3 (C, C-10), 28.2 (C, C-11), 61.3 (C, C-CH_3_-1), 14.1 (C, C-CH_3_-2).

**Javamide-0-propanyl.** NMR data: ^1^H-NMR (*d*_6_-DMSO, 400 MHz) δ 7.58 (1H, d, *J =* 8.2 Hz, H-18), 7.62 (1H, d, *J =* 8.2 Hz, H-1/H-5), 7.38 (1H, t, *J =* 7.3 Hz, H-2/4), 7.37 (1H, d, *J =* 15.6 Hz, H-7), 7.33 (1H, d, *J =* 7.8 Hz, H-15), 7.20 (1H, s, H-13), 7.06 (1H, t, *J =* 7.3 Hz, H-16), 6.98 (1H, t, *J =* 7.3 Hz, H-17), 6.46 (1H, d, *J =* 15.6 Hz, H-8), 4.72 (1H, t, *J =* 7.3 Hz, H-10), 3.24 (1H, dt, *J =* 6.0, 6.9 Hz, H-11), 12.89 (1H, br s, OH-a′), 10.79 (1H, br s, NH-β), 8.38 (1H, br s, NH-α), 4.06 (1H, s, CH_3_-1), 1.73 (1H, s, CH_3_-2), 1.01 (1H, s, CH_3_-3); ^13^C-NMR (*d*_6_-DMSO, 100 MHz) 174.7 (C, C-20), 166.8 (C, C-9), 141.7 (C, C-7), 136.4 (C, C-14), 128.5 (C, C-1/5), 135.2 (C, C-6), 128.6 (C, C-2/4), 127.9 (C, C-3), 127.4 (C, C-19), 124.0 (C, C-8), 123.0 (C, C-13), 121.7 (C, C-16), 119.8 (C, C-17), 118.8 (C, C-18), 111.5 (C, C-15), 109.3 (C, C-12), 60.3 (C, C-10), 28.2 (C, C-11), 66.2 (C, C-CH_3_-1), 21.9 (C, C-CH_3_-2), 10.3 (C, C-CH_3_-3), 14.1 (C, C-CH_3_-2).

**Javamide-I**. NMR data: ^1^H-NMR (*d*_6_-DMSO, 400 MHz) δ 7.58 (1H, d, *J =* 8.2 Hz, H-18), 7.45 (1H, d, *J =* 8.2 Hz, H-1/H-5), 7.37 (1H, d, *J =* 15.6 Hz, H-7), 7.33 (1H, d, *J =* 7.8 Hz, H-15), 7.20 (1H, s, H-13), 7.06 (1H, t, *J =* 7.3 Hz, H-16), 6.98 (1H, t, *J =* 7.3 Hz, H-17), 6.59 (1H, d, *J =* 8.2 Hz, H-2/H-4), 6.46 (1H, d, *J =* 15.6 Hz, H-8), 4.72 (1H, t, *J =* 7.3 Hz, H-10), 3.24 (1H, dt, *J =* 6.0, 6.9 Hz, H-11), 9.68 (1H, br s, OH-a), 12.89 (1H, br s, OH-a′), 10.79 (1H, br s, NH-β), 8.38 (1H, br s, NH-α); ^13^C-NMR (*d*_6_-DMSO, 100 MHz) 174.7 (C, C-20), 166.8 (C, C-9), 157.5 (C, C-3), 141.7 (C, C-7), 136.4 (C, C-14), 130.6 (C, C-1), 130.6 (C, C-5), 127.9 (C, C-6), 127.4 (C, C-19), 124.0 (C, C-8), 123.0 (C, C-13), 121.7 (C, C-16), 119.8 (C, C-17), 118.8 (C, C-18), 115.7 (C, C-2), 115.7 (C, C-4), 111.5 (C, C-15), 109.3 (C, C-12), 60.3 (C, C-10), 28.2 (C, C-11).

**Javamide-I-methyl**. NMR data: ^1^H-NMR (*d*_6_-DMSO, 400 MHz) δ 7.58 (1H, d, *J =* 8.2 Hz, H-18), 7.45 (1H, d, *J =* 8.2 Hz, H-1/H-5), 7.37 (1H, d, *J =* 15.6 Hz, H-7), 7.33 (1H, d, *J =* 7.8 Hz, H-15), 7.20 (1H, s, H-13), 7.06 (1H, t, *J =* 7.3 Hz, H-16), 6.98 (1H, t, *J =* 7.3 Hz, H-17), 6.59 (1H, d, *J =* 8.2 Hz, H-2/H-4), 6.46 (1H, d, *J =* 15.6 Hz, H-8), 4.72 (1H, t, *J =* 7.3 Hz, H-10), 3.24 (1H, dt, *J =* 6.0. 6.9 Hz, H-11), 9.68 (1H, br s, OH-a), 12.89 (1H, br s, OH-a′), 10.79 (1H, br s, NH-β), 8.38 (1H, br s, NH-α), 3.66 (1H, s, CH_3_-1); ^13^C-NMR (*d*_6_-DMSO, 100 MHz) 174.7 (C, C-20), 166.8 (C, C-9), 157.5 (C, C-3), 141.7 (C, C-7), 136.4 (C, C-14), 130.6 (C, C-1), 130.6 (C, C-5), 127.9 (C, C-6), 127.4 (C, C-19), 124.0 (C, C-8), 123.0 (C, C-13), 121.7 (C, C-16), 119.8 (C, C-17), 118.8 (C, C-18), 115.7 (C, C-2), 115.7 (C, C-4), 111.5 (C, C-15), 109.3 (C, C-12), 60.3 (C, C-10), 28.2 (C, C-11), 51.9 (C, C-CH_3_-1).

**Javamide-I-ethyl**. NMR data: ^1^H-NMR (*d*_6_-DMSO, 400 MHz) δ 7.58 (1H, d, *J =* 8.2 Hz, H-18), 7.45 (1H, d, *J =* 8.2 Hz, H-1/H-5), 7.37 (1H, d, *J =* 15.6 Hz, H-7), 7.33 (1H, d, *J =* 7.8 Hz, H-15), 7.20 (1H, s, H-13), 7.06 (1H, t, *J =* 7.3 Hz, H-16), 6.98 (1H, t, *J =* 7.3 Hz, H-17), 6.59 (1H, d, *J =* 8.2 Hz, H-2/H-4), 6.46 (1H, d, *J =* 15.6 Hz, H-8), 4.72 (1H, t, *J =* 7.3 Hz, H-10), 3.24 (1H, dt, *J =* 6.0, 6.9 Hz, H-11), 9.68 (1H, br s, OH-a), 12.89 (1H, br s, OH-a′), 10.79 (1H, br s, NH-β), 8.38 (1H, br s, NH-α), 4.07 (1H, s, CH_3_-1), 1.21 (1H, s, CH_3_-2); ^13^C-NMR (*d*_6_-DMSO, 100 MHz) 174.7 (C, C-20), 166.8 (C, C-9), 157.5 (C, C-3), 141.7 (C, C-7), 136.4 (C, C-14), 130.6 (C, C-1), 130.6 (C, C-5), 127.9 (C, C-6), 127.4 (C, C-19), 124.0 (C, C-8), 123.0 (C, C-13), 121.7 (C, C-16), 119.8 (C, C-17), 118.8 (C, C-18), 115.7 (C, C-2), 115.7 (C, C-4), 111.5 (C, C-15), 109.3 (C, C-12), 60.3 (C, C-10), 28.2 (C, C-11), 61.3 (C, C-CH_3_-1), 14.1 (C, C-CH_3_-2).

**Javamide-I-propanyl**. NMR data: ^1^H-NMR (*d*_6_-DMSO, 400 MHz) δ 7.58 (1H, d, *J =* 8.2 Hz, H-18), 7.45 (1H, d, *J =* 8.2 Hz, H-1/H-5), 7.37 (1H, d, *J =* 15.6 Hz, H-7), 7.33 (1H, d, *J =* 7.8 Hz, H-15), 7.20 (1H, s, H-13), 7.06 (1H, t, *J =* 7.3 Hz, H-16), 6.98 (1H, t, *J =* 7.3 Hz, H-17), 6.59 (1H, d, *J =* 8.2 Hz, H-2/H-4), 6.46 (1H, d, *J =* 15.6 Hz, H-8), 4.72 (1H, t, *J =* 7.3 Hz, H-10), 3.24 (1H, dt, *J =* 6.0, 6.9 Hz, H-11), 9.68 (1H, br s, OH-a), 12.89 (1H, br s, OH-a′), 10.79 (1H, br s, NH-β), 8.38 (1H, br s, NH-α), 4.06 (1H, s, CH_3_-1), 1.73 (1H, s, CH_3_-2), 1.01 (1H, s, CH_3_-3); ^13^C-NMR (*d*_6_-DMSO, 100 MHz) 174.7 (C, C-20), 166.8 (C, C-9), 157.5 (C, C-3), 141.7 (C, C-7), 136.4 (C, C-14), 130.6 (C, C-1), 130.6 (C, C-5), 127.9 (C, C-6), 127.4 (C, C-19), 124.0 (C, C-8), 123.0 (C, C-13), 121.7 (C, C-16), 119.8 (C, C-17), 118.8 (C, C-18), 115.7 (C, C-2), 115.7 (C, C-4), 111.5 (C, C-15), 109.3 (C, C-12), 60.3 (C, C-10), 28.2 (C, C-11), 66.2 (C, C-CH_3_-1), 21.9 (C, C-CH_3_-2), 10.3 (C, C-CH_3_-3). 

**Javamide-II**. NMR data: ^1^H-NMR (*d*_6_-DMSO, 400 MHz) δ 7.58 (1H, d, *J =* 8.2 Hz, H-18), 7.33 (1H, d, *J =* 7.8 Hz, H-15), 7.32 (1H, d, *J =* 15.6 Hz, H-7), 7.20 (1H, s, H-13), 7.06 (1H, t, *J =* 7.3 Hz, H-16), 7.06 (1H, s, H-5), 6.98 (1H, t, *J =* 7.3 Hz, H-17), 6.82 (1H, d, *J =* 8.7 Hz, H-2), 6.67 (1H, dd, *J =* 8.2, 1.4 Hz, H-1), 6.46 (1H, d, *J =* 15.6 Hz, H-8), 4.72 (1H, t, *J =* 7.3 Hz, H-10), 3.24 (1H, dt, *J =* 6.0, 6.9 Hz, H-11), 9.48 (1H, br s, OH-a, b), 12.89 (1H, br s, OH-a′), 10.79 (1H, br s, NH-β), 8.38 (1H, br s, NH-α); ^13^C-NMR (*d*_6_-DMSO, 100 MHz) 174.7 (C, C-20), 166.8 (C, C-9), 146.5 (C, C-3), 145.9 (C, C-4), 141.7 (C, C-7), 136.4 (C, C-14), 128.0 (C, C-6), 127.4 (C, C-19), 124.0 (C, C-8), 123.2 (C, C-1), 123.0 (C, C-13), 121.7 (C, C-16), 119.8 (C, C-17), 118.8 (C, C-18), 117.2 (C, C-2), 115.2 (C, C-5), 111.1 (C, C-15), 109.7 (C, C-12), 60.3 (C, C-10), 28.2 (C, C-11).

**Javamide-II-methyl**. NMR data: ^1^H-NMR (*d*_6_-DMSO, 400 MHz) δ 7.58 (1H, d, *J =* 8.2 Hz, H-18), 7.33 (1H, d, *J =* 7.8 Hz, H-15), 7.32 (1H, d, *J =* 15.6 Hz, H-7), 7.20 (1H, s, H-13), 7.06 (1H, t, *J =* 7.3 Hz, H-16), 7.06 (1H, s, H-5), 6.98 (1H, t, *J =* 7.3 Hz, H-17), 6.82 (1H, d, *J =* 8.7 Hz, H-2), 6.67 (1H, dd, *J =* 8.2, 1.4 Hz, H-1), 6.46 (1H, d, *J =* 15.6 Hz, H-8), 4.72 (1H, t, *J =* 7.3 Hz, H-10), 3.24 (1H, dt, *J =* 6.0, 6.9 Hz, H-11), 9.48 (1H, br s, OH-a, b), 12.89 (1H, br s, OH-a′), 10.79 (1H, br s, NH-β), 8.38 (1H, br s, NH-α), 3.66 (1H, s, CH_3_-1); ^13^C-NMR (*d*_6_-DMSO, 100 MHz) 174.7 (C, C-20), 166.8 (C, C-9), 146.5 (C, C-3), 145.9 (C, C-4), 141.7 (C, C-7), 136.4 (C, C-14), 128.0 (C, C-6), 127.4 (C, C-19), 124.0 (C, C-8), 123.2 (C, C-1), 123.0 (C, C-13), 121.7 (C, C-16), 119.8 (C, C-17), 118.8 (C, C-18), 117.2 (C, C-2), 115.2 (C, C-5), 111.1 (C, C-15), 109.7 (C, C-12), 60.3 (C, C-10), 28.2 (C, C-11), 51.9 (C, C-CH_3_-1). 

**Javamide-II-ethyl**. NMR data: ^1^H-NMR (*d*_6_-DMSO, 400 MHz) δ 7.58 (1H, d, *J =* 8.2 Hz, H-18), 7.33 (1H, d, *J =* 7.8 Hz, H-15), 7.32 (1H, d, *J =* 15.6 Hz, H-7), 7.20 (1H, s, H-13), 7.06 (1H, t, *J =* 7.3 Hz, H-16), 7.06 (1H, s, H-5), 6.98 (1H, t, *J =* 7.3 Hz, H-17), 6.82 (1H, d, *J =* 8.7 Hz, H-2), 6.67 (1H, dd, *J =* 8.2, 1.4 Hz, H-1), 6.46 (1H, d, *J =* 15.6 Hz, H-8), 4.72 (1H, t, *J =* 7.3 Hz, H-10), 3.24 (1H, dt, *J =* 6.0, 6.9 Hz, H-11), 9.48 (1H, br s, OH-a, b), 12.89 (1H, br s, OH-a′), 10.79 (1H, br s, NH-β), 8.38 (1H, br s, NH-α), 4.07 (1H, s, CH_3_-1), 1.21 (1H, s, CH_3_-2); ^13^C-NMR (*d*_6_-DMSO, 100 MHz) 174.7 (C, C-20), 166.8 (C, C-9), 146.5 (C, C-3), 145.9 (C, C-4), 141.7 (C, C-7), 136.4 (C, C-14), 128.0 (C, C-6), 127.4 (C, C-19), 124.0 (C, C-8), 123.2 (C, C-1), 123.0 (C, C-13), 121.7 (C, C-16), 119.8 (C, C-17), 118.8 (C, C-18), 117.2 (C, C-2), 115.2 (C, C-5), 111.1 (C, C-15), 109.7 (C, C-12), 60.3 (C, C-10), 28.2 (C, C-11), 61.3 (C, C-CH_3_-1), 14.1 (C, C-CH_3_-2).

**Javamide-II-propanyl**. NMR data: ^1^H-NMR (*d*_6_-DMSO, 400 MHz) δ 7.58 (1H, d, *J =* 8.2 Hz, H-18), 7.33 (1H, d, *J =* 7.8 Hz, H-15), 7.32 (1H, d, *J =* 15.6 Hz, H-7), 7.20 (1H, s, H-13), 7.06 (1H, t, *J =* 7.3 Hz, H-16), 7.06 (1H, s, H-5), 6.98 (1H, t, *J =* 7.3 Hz, H-17), 6.82 (1H, d, *J =* 8.7 Hz, H-2), 6.67 (1H, dd, *J =* 8.2, 1.4 Hz, H-1), 6.46 (1H, d, *J =* 15.6 Hz, H-8), 4.72 (1H, t, *J =* 7.3 Hz, H-10), 3.24 (1H, dt, *J =* 6.0, 6.9 Hz, H-11), 9.48 (1H, br s, OH-a, b), 12.89 (1H, br s, OH-a′), 10.79 (1H, br s, NH-β), 8.38 (1H, br s, NH-α), 4.06 (1H, s, CH_3_-1), 1.73 (1H, s, CH_3_-2), 1.01 (1H, s, CH_3_-3); ^13^C-NMR (*d*_6_-DMSO, 100 MHz) 174.7 (C, C-20), 166.8 (C, C-9), 146.5 (C, C-3), 145.9 (C, C-4), 141.7 (C, C-7), 136.4 (C, C-14), 128.0 (C, C-6), 127.4 (C, C-19), 124.0 (C, C-8), 123.2 (C, C-1), 123.0 (C, C-13), 121.7 (C, C-16), 119.8 (C, C-17), 118.8 (C, C-18), 117.2 (C, C-2), 115.2 (C, C-5), 111.1 (C, C-15), 109.7 (C, C-12), 60.3 (C, C-10), 28.2 (C, C-11), 66.2 (C, C-CH_3_-1), 21.9 (C, C-CH_3_-2), 10.3 (C, C-CH_3_-3). 

**Javamide-III**. NMR data: ^1^H-NMR (*d*_6_-DMSO, 400 MHz) δ 7.58 (1H, d, *J =* 8.2 Hz, H-18), 7.33 (1H, d, *J =* 7.8 Hz, H-15), 7.32 (1H, d, *J =* 15.6 Hz, H-7), 7.20 (1H, s, H-13), 7.06 (1H, t, *J =* 7.3 Hz, H-16), 7.11 (1H, d, *J =* 15.6 Hz, H-5), 6.98 (1H, t, *J =* 7.3 Hz, H-17), 6.89 (1H, d, *J =* 8.7 Hz, H-2), 6.79 (1H, dd, *J =* 8.2, 1.4 Hz, H-1), 6.46 (1H, d, *J* = 15.6 Hz, H-8), 4.72 (1H, t, *J* = 7.3 Hz, H-10), 3.24 (1H, dt, *J* = 6.0, 6.9 Hz, H-11), 9.48 (1H, br s, OH-a), 12.89 (1H, br s, OH-a′), 10.79 (1H, br s, NH-β), 8.38 (1H, br s, NH-α), 3.83 (1H, s, O-CH_3_); ^13^C-NMR (*d*_6_-DMSO, 100 MHz) 174.7 (C, C-20), 166.8 (C, C-9), 147.9 (C, C-3), 149.1 (C, C-4), 141.7 (C, C-7), 136.4 (C, C-14), 128.0 (C, C-6), 127.4 (C, C-19), 124.0 (C, C-8), 123.2 (C, C-1), 123.0 (C, C-13), 121.7 (C, C-16), 119.8 (C, C-17), 118.8 (C, C-18), 117.2 (C, C-2), 115.2 (C, C-5), 111.1 (C, C-15), 109.7 (C, C-12), 60.3 (C, C-10), 28.2 (C, C-11), 56.1 (C, -CH_3_-O-b). 

**Javamide-III-methyl**. NMR data: ^1^H-NMR (*d*_6_-DMSO, 400 MHz) δ 7.58 (1H, d, *J =* 8.2 Hz, H-18), 7.33 (1H, d, *J =* 7.8 Hz, H-15), 7.32 (1H, d, *J =* 15.6 Hz, H-7), 7.20 (1H, s, H-13), 7.06 (1H, t, *J =* 7.3 Hz, H-16), 7.11 (1H, d, *J =* 15.6 Hz, H-5), 6.98 (1H, t, *J =* 7.3 Hz, H-17), 6.89 (1H, d, *J =* 8.7 Hz, H-2), 6.79 (1H, dd, *J =* 8.2, 1.4 Hz, H-1), 6.46 (1H, d, *J =* 15.6 Hz, H-8), 4.72 (1H, t, *J =* 7.3 Hz, H-10), 3.24 (1H, dt, *J =* 6.0, 6.9 Hz, H-11), 9.48 (1H, br s, OH-a), 12.89 (1H, br s, OH-a′), 10.79 (1H, br s, NH-β), 8.38 (1H, br s, NH-α), 3.83 (1H, s, OCH_3_-b), 3.66 (1H, s, CH_3_-1); ^13^C-NMR (*d*_6_-DMSO, 100 MHz) 174.7 (C, C-20), 166.8 (C, C-9), 147.9 (C, C-3), 149.1 (C, C-4), 141.7 (C, C-7), 136.4 (C, C-14), 128.0 (C, C-6), 127.4 (C, C-19), 124.0 (C, C-8), 123.2 (C, C-1), 123.0 (C, C-13), 121.7 (C, C-16), 119.8 (C, C-17), 118.8 (C, C-18), 117.2 (C, C-2), 115.2 (C, C-5), 111.1 (C, C-15), 109.7 (C, C-12), 60.3 (C, C-10), 28.2 (C, C-11), 56.1 (C, -CH_3_-O-b), 51.9 (C, C-CH_3_-1).

**Javamide-III-ethyl**. NMR data: ^1^H-NMR (*d*_6_-DMSO, 400 MHz) δ 7.58 (1H, d, *J =* 8.2 Hz, H-18), 7.33 (1H, d, *J =* 7.8 Hz, H-15), 7.32 (1H, d, *J =* 15.6 Hz, H-7), 7.20 (1H, s, H-13), 7.06 (1H, t, *J =* 7.3 Hz, H-16), 7.11 (1H, d, *J =* 15.6 Hz, H-5), 6.98 (1H, t, *J =* 7.3 Hz, H-17), 6.89 (1H, d, *J =* 8.7 Hz, H-2), 6.79 (1H, dd, *J =* 8.2, 1.4 Hz, H-1), 6.46 (1H, d, *J =* 15.6 Hz, H-8), 4.72 (1H, t, *J =* 7.3 Hz, H-10), 3.24 (1H, dt, *J =* 6.0, 6.9 Hz, H-11), 9.48 (1H, br s, OH-a), 12.89 (1H, br s, OH-a′), 10.79 (1H, br s, NH-β), 8.38 (1H, br s, NH-α), 3.83 (1H, s, OCH_3_-b), 4.07 (1H, s, CH_3_-1), 1.21 (1H, s, CH_3_-2); ^13^C-NMR (*d*_6_-DMSO, 100 MHz) 174.7 (C, C-20), 166.8 (C, C-9), 147.9 (C, C-3), 149.1 (C, C-4), 141.7 (C, C-7), 136.4 (C, C-14), 128.0 (C, C-6), 127.4 (C, C-19), 124.0 (C, C-8), 123.2 (C, C-1), 123.0 (C, C-13), 121.7 (C, C-16), 119.8 (C, C-17), 118.8 (C, C-18), 117.2 (C, C-2), 115.2 (C, C-5), 111.1 (C, C-15), 109.7 (C, C-12), 60.3 (C, C-10), 28.2 (C, C-11), 56.1 (C, -CH_3_-O-b), 61.3 (C, C-CH_3_-1), 14.1 (C, C-CH_3_-2).

**Javamide-III-propanyl**. NMR data: ^1^H-NMR (*d*_6_-DMSO, 400 MHz) δ 7.58 (1H, d, *J =* 8.2 Hz, H-18), 7.33 (1H, d, *J =* 7.8 Hz, H-15), 7.32 (1H, d, *J =* 15.6 Hz, H-7), 7.20 (1H, s, H-13), 7.06 (1H, t, *J =* 7.3 Hz, H-16), 7.11 (1H, d, *J =* 15.6 Hz, H-5), 6.98 (1H, t, *J =* 7.3 Hz, H-17), 6.89 (1H, d, *J =* 8.7 Hz, H-2), 6.79 (1H, dd, *J =* 8.2, 1.4 Hz, H-1), 6.46 (1H, d, *J =* 15.6 Hz, H-8), 4.72 (1H, t, *J =* 7.3 Hz, H-10), 3.24 (1H, dt, *J =* 6.0, 6.9 Hz, H-11), 9.48 (1H, br s, OH-a), 12.89 (1H, br s, OH-a′), 10.79 (1H, br s, NH-β), 8.38 (1H, br s, NH-α), 3.83 (1H, s, OCH_3_-b), 4.06 (1H, s, CH_3_-1), 1.73 (1H, s, CH_3_-2), 1.01 (1H, s, CH_3_-3); ^13^C-NMR (*d*_6_-DMSO, 100 MHz) 174.7 (C, C-20), 166.8 (C, C-9), 147.9 (C, C-3), 149.1 (C, C-4), 141.7 (C, C-7), 136.4 (C, C-14), 128.0 (C, C-6), 127.4 (C, C-19), 124.0 (C, C-8), 123.2 (C, C-1), 123.0 (C, C-13), 121.7 (C, C-16), 119.8 (C, C-17), 118.8 (C, C-18), 117.2 (C, C-2), 115.2 (C, C-5), 111.1 (C, C-15), 109.7 (C, C-12), 60.3 (C, C-10), 28.2 (C, C-11), 56.1 (C, -CH_3_-O-b), 66.2 (C, C-CH_3_-1), 21.9 (C, C-CH_3_-2), 10.3 (C, C-CH_3_-3). 

**Javamide-IV**. NMR data: ^1^H-NMR (*d*_6_-DMSO, 400 MHz) δ 7.58 (1H, d, *J =* 8.2 Hz, H-18), 7.33 (1H, d, *J =* 7.8 Hz, H-15), 7.37 (1H, d, *J =* 15.6 Hz, H-7), 7.20 (1H, s, H-13), 7.06 (1H, t, *J =* 7.3 Hz, H-16), 6.74 (1H, s, H-1/5), 6.98 (1H, t, *J =* 7.3 Hz, H-17), 6.46 (1H, d, *J =* 15.6 Hz, H-8), 4.68 (1H, t, *J =* 7.3 Hz, H-10), 3.24 (1H, dt, *J =* 6.0, 6.9 Hz, H-11), 8.73 (1H, br s, OH-a), 12.89 (1H, br s, OH-a′), 10.79 (1H, br s, NH-β), 8.38 (1H, br s, NH-α), 3.83 (1H, s, O-CH_3_-b, c); ^13^C-NMR (*d*_6_-DMSO, 100 MHz) 171.5 (C, C-20), 166.8 (C, C-9), 148.1 (C, C-2/4), 141.7 (C, C-7), 136.6 (C, C-3), 136.4 (C, C-14), 130.6 (C, C-6), 127.4 (C, C-19), 124.0 (C, C-8), 123.0 (C, C-13), 121.7 (C, C-16), 119.8 (C, C-17), 118.8 (C, C-18), 111.1 (C, C-15), 109.7 (C, C-12), 107.2 (C, C-1/5), 59.1 (C, C-10), 28.2 (C, C-11), 56.1 (C, O-CH_3_-b, c).

**Javamide-IV-methyl**. NMR data: ^1^H-NMR (*d*_6_-DMSO, 400 MHz) δ 7.58 (1H, d, *J =* 8.2 Hz, H-18), 7.33 (1H, d, *J =* 7.8 Hz, H-15), 7.37 (1H, d, *J =* 15.6 Hz, H-7), 7.20 (1H, s, H-13), 7.06 (1H, t, *J =* 7.3 Hz, H-16), 6.74 (1H, s, H-1/5), 6.98 (1H, t, *J =* 7.3 Hz, H-17), 6.46 (1H, d, *J =* 15.6 Hz, H-8), 4.68 (1H, t, *J =* 7.3 Hz, H-10), 3.24 (1H, dt, *J =* 6.0, 6.9 Hz, H-11), 8.73 (1H, br s, OH-a), 12.89 (1H, br s, OH-a′), 10.79 (1H, br s, NH-β), 8.38 (1H, br s, NH-α), 3.83 (1H, s, O-CH_3_-b, c), 3.66 (1H, s, CH_3_-1); ^13^C-NMR (*d*_6_-DMSO, 100 MHz) 171.5 (C, C-20), 166.8 (C, C-9), 148.1 (C, C-2/4), 141.7 (C, C-7), 136.6 (C, C-3), 136.4 (C, C-14), 130.6 (C, C-6), 127.4 (C, C-19), 124.0 (C, C-8), 123.0 (C, C-13), 121.7 (C, C-16), 119.8 (C, C-17), 118.8 (C, C-18), 111.1 (C, C-15), 109.7 (C, C-12), 107.2 (C, C-1/5), 59.1 (C, C-10), 28.2 (C, C-11), 56.1 (C, O-CH_3_-b, c), 51.9 (C, C-CH_3_-1).

**Javamide-IV-ethyl**. NMR data: ^1^H-NMR (*d*_6_-DMSO, 400 MHz) δ 7.58 (1H, d, *J =* 8.2 Hz, H-18), 7.33 (1H, d, *J =* 7.8 Hz, H-15), 7.37 (1H, d, *J =* 15.6 Hz, H-7), 7.20 (1H, s, H-13), 7.06 (1H, t, *J =* 7.3 Hz, H-16), 6.74 (1H, s, H-1/5), 6.98 (1H, t, *J =* 7.3 Hz, H-17), 6.46 (1H, d, *J =* 15.6 Hz, H-8), 4.68 (1H, t, *J =* 7.3 Hz, H-10), 3.24 (1H, dt, *J =* 6.0, 6.9 Hz, H-11), 8.73 (1H, br s, OH-a), 12.89 (1H, br s, OH-a′), 10.79 (1H, br s, NH-β), 8.38 (1H, br s, NH-α), 3.83 (1H, s, O-CH_3_-b, c), 4.07 (1H, s, CH_3_-1), 1.21 (1H, s, CH_3_-2); ^13^C-NMR (*d*_6_-DMSO, 100 MHz) 171.5 (C, C-20), 166.8 (C, C-9), 148.1 (C, C-2/4), 141.7 (C, C-7), 136.6 (C, C-3), 136.4 (C, C-14), 130.6 (C, C-6), 127.4 (C, C-19), 124.0 (C, C-8), 123.0 (C, C-13), 121.7 (C, C-16), 119.8 (C, C-17), 118.8 (C, C-18), 111.1 (C, C-15), 109.7 (C, C-12), 107.2 (C, C-1/5), 59.1 (C, C-10), 28.2 (C, C-11), 56.1 (C, O-CH_3_-b, c), 61.3 (C, C-CH_3_-1), 14.1 (C, C-CH_3_-2).

**Javamide-IV-propanyl**. NMR data: ^1^H-NMR (*d*_6_-DMSO, 400 MHz) δ 7.58 (1H, d, *J =* 8.2 Hz, H-18), 7.33 (1H, d, *J =* 7.8 Hz, H-15), 7.37 (1H, d, *J =* 15.6 Hz, H-7), 7.20 (1H, s, H-13), 7.06 (1H, t, *J =* 7.3 Hz, H-16), 6.74 (1H, s, H-1/5), 6.98 (1H, t, *J =* 7.3 Hz, H-17), 6.46 (1H, d, *J =* 15.6 Hz, H-8), 4.68 (1H, t, *J =* 7.3 Hz, H-10), 3.24 (1H, dt, *J =* 6.0, 6.9 Hz, H-11), 8.73 (1H, br s, OH-a), 12.89 (1H, br s, OH-a′), 10.79 (1H, br s, NH-β), 8.38 (1H, br s, NH-α), 3.83 (1H, s, O-CH_3_-b, c), 4.06 (1H, s, CH_3_-1), 1.73 (1H, s, CH_3_-2), 1.01 (1H, s, CH_3_-3); ^13^C-NMR (*d*_6_-DMSO, 100 MHz) 171.5 (C, C-20), 166.8 (C, C-9), 148.1 (C, C-2/4), 141.7 (C, C-7), 136.6 (C, C-3), 136.4 (C, C-14), 130.6 (C, C-6), 127.4 (C, C-19), 124.0 (C, C-8), 123.0 (C, C-13), 121.7 (C, C-16), 119.8 (C, C-17), 118.8 (C, C-18), 111.1 (C, C-15), 109.7 (C, C-12), 107.2 (C, C-1/5), 59.1 (C, C-10), 28.2 (C, C-11), 56.1 (C, O-CH_3_-b, c), 66.2 (C, C-CH_3_-1), 21.9 (C, C-CH_3_-2), 10.3 (C, C-CH_3_-3).

**Safflomide-0**. NMR data: ^1^H-NMR (*d*_6_-DMSO, 400 MHz) δ 7.58 (1H, d, *J =* 8.2 Hz, H-18), 7.62 (1H, d, *J =* 8.2 Hz, H-1/H-5), 7.38 (1H, t, *J =* 7.3 Hz, H-2/4), 7.37 (1H, d, *J =* 15.6 Hz, H-7), 7.33 (1H, d, *J =* 7.8 Hz, H-15), 7.20 (1H, s, H-13), 7.06 (1H, t, *J =* 7.3 Hz, H-16), 6.98 (1H, t, *J =* 7.3 Hz, H-17), 6.46 (1H, d, *J =* 15.6 Hz, H-8), 3.49 (1H, t, *J =* 7.3 Hz, H-10), 2.86 (1H, dt, *J =* 6.0, 6.9 Hz, H-11), 10.79 (1H, br s, NH-β), 8.41 (1H, br s, NH-α); ^13^C-NMR (*d*_6_-DMSO, 100 MHz) 166.8 (C, C-9), 141.7 (C, C-7), 136.4 (C, C-14), 128.5 (C, C-1/5), 135.2 (C, C-6), 128.6 (C, C-2/4), 127.9 (C, C-3), 127.4 (C, C-19), 124.0 (C, C-8), 123.0 (C, C-13), 121.7 (C, C-16), 119.8 (C, C-17), 118.8 (C, C-18), 111.5 (C, C-15), 113.0 (C, C-12), 41.7 (C, C-10), 25.5 (C, C-11).

**Safflomide-I**. NMR data: ^1^H-NMR (*d*_6_-DMSO, 400 MHz) δ 7.58 (1H, d, *J =* 8.2 Hz, H-18), 7.45 (1H, d, *J =* 8.2 Hz, H-1/H-5), 7.37 (1H, d, *J =* 15.6 Hz, H-7), 7.33 (1H, d, *J =* 7.8 Hz, H-15), 7.20 (1H, s, H-13), 7.06 (1H, t, *J =* 7.3 Hz, H-16), 6.98 (1H, t, *J =* 7.3 Hz, H-17), 6.59 (1H, d, *J =* 8.2 Hz, H-2/H-4), 6.46 (1H, d, *J =* 15.6 Hz, H-8), 3.49 (1H, t, *J =* 7.3 Hz, H-10), 2.86 (1H, dt, *J =* 6.0, 6.9 Hz, H-11), 9.68 (1H, br s, OH-a), 12.89 (1H, br s, OH-a′), 10.79 (1H, br s, NH-β), 8.41 (1H, br s, NH-α); ^13^C-NMR (*d*_6_-DMSO, 100 MHz) 166.8 (C, C-9), 157.5 (C, C-3), 141.7 (C, C-7), 136.4 (C, C-14), 130.6 (C, C-1), 130.6 (C, C-5), 127.9 (C, C-6), 127.4 (C, C-19), 124.0 (C, C-8), 123.0 (C, C-13), 121.7 (C, C-16), 119.8 (C, C-17), 118.8 (C, C-18), 115.7 (C, C-2), 115.7 (C, C-4), 111.5 (C, C-15), 113.0 (C, C-12), 41.7 (C, C-10), 25.5 (C, C-11).

**Safflomide-II**. NMR data: ^1^H-NMR (*d*_6_-DMSO, 400 MHz) δ 7.58 (1H, d, *J =* 8.2 Hz, H-18), 7.33 (1H, d, *J =* 7.8 Hz, H-15), 7.32 (1H, d, *J =* 15.6 Hz, H-7), 7.20 (1H, s, H-13), 7.06 (1H, t, *J =* 7.3 Hz, H-16), 7.06 (1H, s, H-5), 6.98 (1H, t, *J =* 7.3 Hz, H-17), 6.82 (1H, d, *J =* 8.7 Hz, H-2), 6.67 (1H, dd, *J =* 8.2, 1.4 Hz, H-1), 6.46 (1H, d, *J =* 15.6 Hz, H-8), 3.49 (1H, t, *J =* 7.3 Hz, H-10), 2.86 (1H, dt, *J =* 6.0, 6.9 Hz, H-11), 9.48 (1H, br s, OH-a, b), 10.79 (1H, br s, NH-β), 8.41 (1H, br s, NH-α); ^13^C-NMR (*d*_6_-DMSO, 100 MHz) 166.8 (C, C-9), 146.5 (C, C-3), 145.9 (C, C-4), 141.7 (C, C-7), 136.4 (C, C-14), 128.0 (C, C-6), 127.4 (C, C-19), 124.0 (C, C-8), 123.2 (C, C-1), 123.0 (C, C-13), 121.7 (C, C-16), 119.8 (C, C-17), 118.8 (C, C-18), 117.2 (C, C-2), 115.2 (C, C-5), 111.1 (C, C-15), 113.0 (C, C-12), 41.7 (C, C-10), 25.5 (C, C-11).

**Safflomide-III**. NMR data: ^1^H-NMR (*d*_6_-DMSO, 400 MHz) δ 7.58 (1H, d, *J =* 8.2 Hz, H-18), 7.33 (1H, d, *J =* 7.8 Hz, H-15), 7.32 (1H, d, *J =* 15.6 Hz, H-7), 7.20 (1H, s, H-13), 7.06 (1H, t, *J =* 7.3 Hz, H-16), 7.11 (1H, d, *J =* 15.6 Hz, H-5), 6.98 (1H, t, *J =* 7.3 Hz, H-17), 6.89 (1H, d, *J =* 8.7 Hz, H-2), 6.79 (1H, dd, *J =* 8.2, 1.4 Hz, H-1), 6.46 (1H, d, *J =* 15.6 Hz, H-8), 3.49 (1H, t, *J =* 7.3 Hz, H-10), 2.86 (1H, dt, *J =* 6.0, 6.9 Hz, H-11), 9.48 (1H, br s, OH-a), 10.79 (1H, br s, NH-β), 8.41 (1H, br s, NH-α), 3.83 (1H, s, O-CH_3_); ^13^C-NMR (*d*_6_-DMSO, 100 MHz) 174.7 (C, C-20), 166.8 (C, C-9), 147.9 (C, C-3), 149.1 (C, C-4), 141.7 (C, C-7), 136.4 (C, C-14), 128.0 (C, C-6), 127.4 (C, C-19), 124.0 (C, C-8), 123.2 (C, C-1), 123.0 (C, C-13), 121.7 (C, C-16), 119.8 (C, C-17), 118.8 (C, C-18), 117.2 (C, C-2), 115.2 (C, C-5), 111.1 (C, C-15), 113.0 (C, C-12), 41.7 (C, C-10), 25.5 (C, C-11), 56.1 (C, -CH_3_-O-b).

**Safflomide-IV**. NMR data: NMR data: ^1^H-NMR (*d*_6_-DMSO, 400 MHz) δ 7.58 (1H, d, *J =* 8.2 Hz, H-18), 7.33 (1H, d, *J* = 7.8 Hz, H-15), 7.37 (1H, d, *J =* 15.6 Hz, H-7), 7.20 (1H, s, H-13), 7.06 (1H, t, *J =* 7.3 Hz, H-16), 6.74 (1H, s, H-1/5), 6.98 (1H, t, *J =* 7.3 Hz, H-17), 6.46 (1H, d, *J =* 15.6 Hz, H-8), 3.49 (1H, t, *J =* 7.3 Hz, H-10), 2.864 (1H, dt, *J =* 6.0, 6.9 Hz, H-11), 8.73 (1H, br s, OH-a), 10.79 (1H, br s, NH-β), 8.38 (1H, br s, NH-α), 3.83 (1H, s, OH-b, c); ^13^C-NMR (*d*_6_-DMSO, 100 MHz) 166.8 (C, C-9), 148.1 (C, C-2/4), 141.7 (C, C-7), 136.6 (C, C-3), 136.4 (C, C-14), 130.6 (C, C-6), 127.4 (C, C-19), 124.0 (C, C-8), 123.0 (C, C-13), 121.7 (C, C-16), 119.8 (C, C-17), 118.8 (C, C-18), 111.1 (C, C-15), 107.2 (C, C-1/5), 113.0 (C, C-12), 41.7 (C, C-10), 25.5 (C, C-11), 56.1 (C, O-CH_3_-b, c).

**Serotomide-0**. NMR data: ^1^H-NMR (*d*_6_-DMSO, 400 MHz) δ 6.94 (1H, d, *J* = 8.2 Hz, H-18), 7.62 (1H, d, *J* = 8.2 Hz, H-1/H-5), 7.38 (1H, t, *J* = 7.3 Hz, H-2/4), 7.37 (1H, d, *J* = 15.6 Hz, H-7), 7.05 (1H, d, *J* = 7.8 Hz, H-15), 7.77 (1H, s, H-13), 6.64 (1H, t, *J* = 7.3 Hz, H-16), 6.46 (1H, d, *J* = 15.6 Hz, H-8), 3.49 (1H, t, *J* = 7.3 Hz, H-10), 2.86 (1H, dt, *J* = 6.0, 6.9 Hz, H-11), 10.79 (1H, br s, NH-β), 8.41 (1H, br s, NH-α), 10.07 (1H, s, indol-OH); ^13^C-NMR (*d*_6_-DMSO, 100 MHz) 166.8 (C, C-9), 141.7 (C, C-7), 131.9 (C, C-14), 128.5 (C, C-1/5), 135.2 (C, C-6), 128.6 (C, C-2/4), 127.9 (C, C-3), 128.8 (C, C-19), 124.0 (C, C-8), 123.0 (C, C-13), 112.7 (C, C-16), 152.4 (C, C-17), 103.6 (C, C-18), 112.5 (C, C-15), 113.0 (C, C-12), 41.7 (C, C-10), 25.5 (C, C-11).

**Serotomide-I**. NMR data: ^1^H-NMR (*d*_6_-DMSO, 400 MHz) δ 7.58 (1H, d, *J* = 8.2 Hz, H-18), 7.45 (1H, d, *J* = 8.2 Hz, H-1/H-5), 7.37 (1H, d, *J* = 15.6 Hz, H-7), 7.33 (1H, d, *J* = 7.8 Hz, H-15), 7.77 (1H, s, H-13), 6.64 (1H, t, *J* = 7.3 Hz, H-16), 6.59 (1H, d, *J* = 8.2 Hz, H-2/H-4), 6.46 (1H, d, *J* = 15.6 Hz, H-8), 3.49 (1H, t, *J* = 7.3 Hz, H-10), 2.86 (1H, dt, *J* = 6.0, 6.9 Hz, H-11), 9.68 (1H, br s, OH-a), 12.89 (1H, br s, OH-a′), 10.79 (1H, br s, NH-β), 8.41 (1H, br s, NH-α), 10.07 (1H, s, indol-OH); ^13^C-NMR (*d*_6_-DMSO, 100 MHz) 166.8 (C, C-9), 157.5 (C, C-3), 141.7 (C, C-7), 131.9 (C, C-14), 130.6 (C, C-1), 130.6 (C, C-5), 127.9 (C, C-6), 128.8 (C, C-19), 124.0 (C, C-8), 123.0 (C, C-13), 112.7 (C, C-16), 152.4 (C, C-17), 103.6 (C, C-18), 115.7 (C, C-2), 115.7 (C, C-4), 112.5 (C, C-15), 113.0 (C, C-12), 41.7 (C, C-10), 25.5 (C, C-11).

**Serotomide-II**. NMR data: ^1^H-NMR (*d*_6_-DMSO, 400 MHz) δ 6.94 (1H, d, *J* = 8.2 Hz, H-18), 7.05 (1H, d, *J* = 7.8 Hz, H-15), 7.32 (1H, d, *J* = 15.6 Hz, H-7), 7.77 (1H, s, H-13), 6.64 (1H, t, *J* = 7.3 Hz, H-16), 7.06 (1H, s, H-5), 6.82 (1H, d, *J* = 8.7 Hz, H-2), 6.67 (1H, dd, *J* = 8.2, 1.4 Hz, H-1), 6.46 (1H, d, *J* = 15.6 Hz, H-8), 3.49 (1H, t, *J* = 7.3 Hz, H-10), 2.86 (1H, dt, *J* = 6.0, 6.9 Hz, H-11), 9.48 (1H, br s, OH-a, b), 10.79 (1H, br s, NH-β), 8.41 (1H, br s, NH-α), 10.07 (1H, s, indol-OH); ^13^C-NMR (*d*_6_-DMSO, 100 MHz) 166.8 (C, C-9), 146.5 (C, C-3), 145.9 (C, C-4), 141.7 (C, C-7), 136.4 (C, C-14), 128.0 (C, C-6), 128.8 (C, C-19), 124.0 (C, C-8), 123.2 (C, C-1), 123.0 (C, C-13), 112.7 (C, C-16), 152.4 (C, C-17), 103.6 (C, C-18), 117.2 (C, C-2), 115.2 (C, C-5), 112.5 (C, C-15), 113.0 (C, C-12), 41.7 (C, C-10), 25.5 (C, C-11).

**Serotomide-III**. NMR data: ^1^H-NMR (*d*_6_-DMSO, 400 MHz) δ 6.94 (1H, d, *J* = 8.2 Hz, H-18), 7.05 (1H, d, *J* = 7.8 Hz, H-15), 7.32 (1H, d, *J* = 15.6 Hz, H-7), 7.77 (1H, s, H-13), 6.64 (1H, t, *J* = 7.3 Hz, H-16), 7.11 (1H, d, *J* = 15.6 Hz, H-5), 6.89 (1H, d, *J* = 8.7 Hz, H-2), 6.79 (1H, dd, *J* = 8.2, 1.4 Hz, H-1), 6.46 (1H, d, *J* = 15.6 Hz, H-8), 3.49 (1H, t, *J* = 7.3 Hz, H-10), 2.86 (1H, dt, *J* = 6.0, 6.9 Hz, H-11), 9.48 (1H, br s, OH-a), 10.79 (1H, br s, NH-β), 8.41 (1H, br s, NH-α), 3.83 (1H, s, O-CH_3_), 10.07 (1H, s, indol-OH); ^13^C-NMR (*d*_6_-DMSO, 100 MHz) 174.7 (C, C-20), 166.8 (C, C-9), 147.9 (C, C-3), 149.1 (C, C-4), 141.7 (C, C-7), 136.4 (C, C-14), 128.0 (C, C-6), 128.8 (C, C-19), 124.0 (C, C-8), 123.2 (C, C-1), 123.0 (C, C-13), 112.7 (C, C-16), 152.4 (C, C-17), 103.6 (C, C-18), 117.2 (C, C-2), 115.2 (C, C-5), 112.5 (C, C-15), 113.0 (C, C-12), 41.7 (C, C-10), 25.5 (C, C-11), 56.1 (C, -CH_3_-O-b).

**Serotomide-IV**. NMR data: ^1^H-NMR (*d*_6_-DMSO, 400 MHz) δ 6.94 (1H, d, *J* = 8.2 Hz, H-18), 7.05 (1H, d, *J* = 7.8 Hz, H-15), 7.37 (1H, d, *J* = 15.6 Hz, H-7), 7.77 (1H, s, H-13), 6.64 (1H, t, *J* = 7.3 Hz, H-16), 6.74 (1H, s, H-1/5), 6.46 (1H, d, *J* = 15.6 Hz, H-8), 3.49 (1H, t, *J* = 7.3 Hz, H-10), 2.864 (1H, dt, *J* = 6.0, 6.9 Hz, H-11), 8.73 (1H, br s, OH-a), 10.79 (1H, br s, NH-β), 8.38 (1H, br s, NH-α), 3.83 (1H, s, OH-b, c), 10.07 (1H, s, indol-OH); ^13^C-NMR (*d*_6_-DMSO, 100 MHz) 166.8 (C, C-9), 148.1 (C, C-2/4), 141.7 (C, C-7), 136.6 (C, C-3), 131.9 (C, C-14), 130.6 (C, C-6), 128.8 (C, C-19), 124.0 (C, C-8), 123.0 (C, C-13), 112.5 (C, C-16), 152.4 (C, C-17), 103.6 (C, C-18), 112.5 (C, C-15), 107.2 (C, C-1/5), 113.0 (C, C-12), 41.7 (C, C-10), 25.5 (C, C-11), 56.1 (C, O-CH_3_-b, c).

### 2.2. In Silico Screening

**Figure 2:**
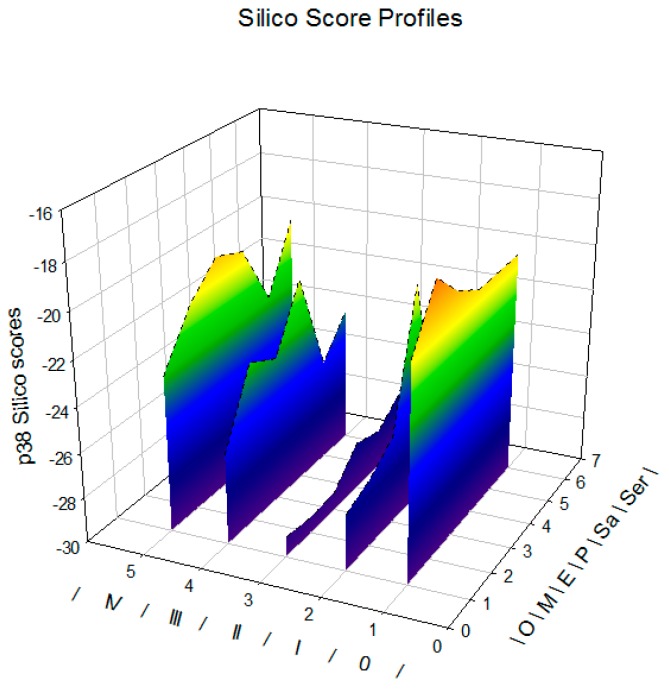
In silico score profiling. 0, I, II, III, and IV axis represented each chemical group and O, M, E, P, Sa, and Sr axis represented parent compounds, methyl, ethyl, propanyl esters, safflomides, serotomides.

In silico screening was performed based on structure-based design (SBD) approach using an algorithm-based docking program ICM-pro as described in “Materials and Methods”. The docking experiments were performed using thirty javamide analogues on the experimental co-crystallized p38 MAP kinase complexes (14 complexes) and the resulting lowest energy and other scoring values were compared (Table 1). For predicting potential active sites, most probable binding site and sub binding sites for javamide analogues were calculated using ICM-pro as described in its manual. ICM-pro predicts potential binding pockets in terms of calculated descriptors (geometrical and physicochemical properties). In general, candidate compounds form favorable interactions with the binding site with largest interacting cavity, so active pocket #1 can be designated as a most potential binding pocket for tested compounds. After finding a most potential binding pocket for tested compounds, the p38 MAPK complex was divided into ligand and protein, and javamide analogues were individually docked into the potential binding pocket of each p38 MAPK complex. For this study, javamide analogues were divided into seven groups; javamide-0/-I/-II/-III/-IV, safflomide and serotomide, and the each group has several derivatives as shown in Figure 1. Each derivative in the seven groups was placed in the most favorite pocket and their binding energy was calculated. The scores and ΔE values of the first two derivatives (javamide-0 and javamide-0-methyl) were shown in Table 1. Similarly, the rest of javamide analogues was analyzed to provide their scores and ΔE values (Table 2). The order of the best scores was Javamide-II-methyl > Javamide-II-ethyl > Javamide-II > Safflomide-II > Javamide-I (Table 2 and Figure 2). As noticed, three highest scores were from javamide-II analogues, suggesting the chemical structure of javamide-II may be preferably accessible to the tested pocket of p38 MAPK complexes.

### 2.3. The Comparison of In Silico Screening of Javamide-II-Methyl Ester and SB 202190

**Figure 3:**
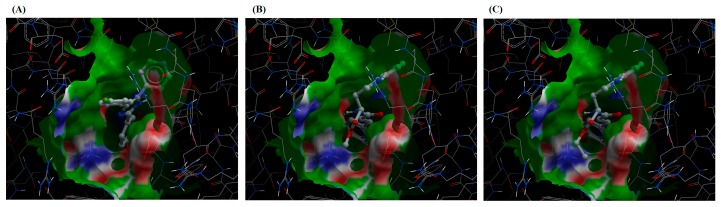
The docking of SB202190, javamide-II-methyl ester and javamide-II-ethyl ester. SB202190 (**A**) javamide-I-methyl ester (**B**) and ethyl ester (**C**) to p38 complex (3HV5) was presented with two potential hydrogen bonds denoted by two green dots (E71) and (D168).

Since javamide-II-methyl was found with the best score, the analogue was comparatively analyzed with a p38 inhibitor (SB 202190). The binding energy (ΔE) of javamide-II-methyl and SB 202190 were −39.1 ± 4.5 and −28.3 ± 6.4, respectively, showing that the analogues may bind to the putative pocket stronger than SB 202190 (Table 3). Also, the data of molecular docking of javamide-II-methyl showed two potential hydrogen bonds which are missing in that of SB 202190, meanwhile javamide-II-ethyl (the second best score) maintained the two hydrogen bonds (Figure 3). Altogether, the docking data showed that javamide-II-methyl and -ethyl could have lower energy and scoring values than SB 202190, suggesting that the analogues may inhibit p38 MAPK better than SB 202190.

### 2.4. p38 MAPK Inhibition Assay

**Figure 4:**
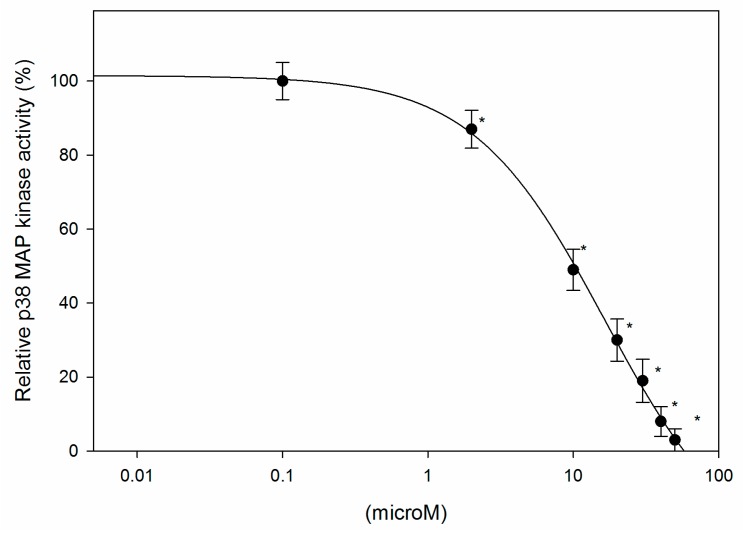
The inhibition of p38 α and determination of IC_50_. The inhibition of p38 α by javamide-II-ethyl ester was determined at the concentrations of 0, 2, 10, 20, 30, 40 and 50 μM. Data points are shown as the means ± S.D. (*n* = 4). The *p* value was calculated using one-way ANOVA with the Bonferroni’s method, and the asterisks (*) represent significant differences (*p* < 0.05) from the vehicle control.

As discussed previously, the p38 MAPK pathway is deeply involved in the production of inflammatory chemokines such as TNF-α, IL-8, RANTES, and MCP-1 [19,20]. Among p38 MAPK isoforms (α, β, γ, Δ), p38 α is believed to be critically involved in cytokine production and inflammatory process [19]. Therefore, the effects of javamide analogues on p38 α were investigated using the CMGC-1 kinase selectivity profiling system (Promega, Madison, WI, USA) as described in “Materials and Methods”. As shown in Table 4, some analogues were able to inhibit p38 α to great extents; the decreasing order of the inhibition was javamide-II-ethyl > javamide-II-methyl > javamide-II > safflomide-II > javamide-III. Surprisingly, the data showed that three analogues out of the five analogues with best docking scores were found to inhibit p38 α significantly (31–33%) and all top three chemicals were javamide-II analogues. Since javamide-II-ethyl was the best inhibitor in vitro p38 MAPK assay, the IC_50_ for p38 α was determined at the concentrations between 0–50 μM. The inhibition was nearly complete at the concentrations higher than 50 μM, and the IC_50_ value for p38 α was found to be 9.9 μM (Figure 4). The data suggest that javamide-II-ethyl may be a potent p38 MAPK inhibitor affecting downstream molecules involved in imitation/progression of inflammation.

### 2.5. The Comparison of In Silico Screening with In Vitro p38 MAPK Inhibition

Because some analogues with best docking scores were also found to have strong p38 MAPK inhibition activity, the data of in silico screening and in vitro p38 MAPK inhibition were compared. As shown in Table 5, top three javamide analogues were selected from both experiments, exhibiting a low range of binding score (ΔE = −39.0 to −39.1) as well as inhibiting p38 MAPK strongly, although there is a slight difference in efficacy sequence. Nonetheless, the data showed a positive correlation between in silico modeling scores and p38 inhibition.

### 2.6. Cytotoxicity Assay

**Figure 5:**
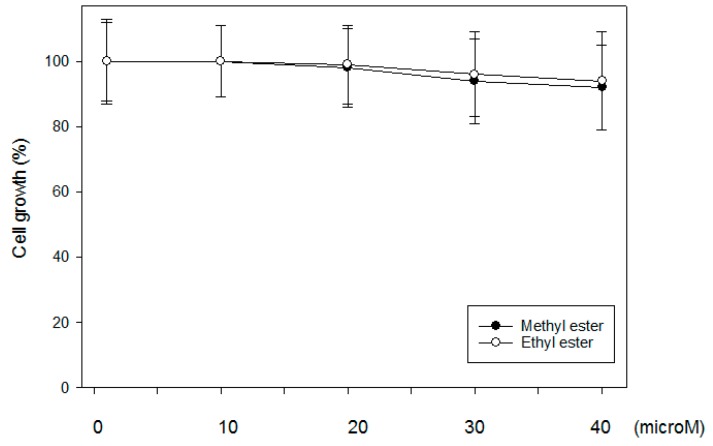
Effects of javamide-II-methyl and -ethyl esters on cell death. The THP-1 cells (1 × 10^6^ cells/well) plated in 12-well plates were treated with 0, 10, 20, 30 and 40 μM of both esters for 18 h. Cell death was analyzed as described previously [16].

As mentioned previously, one of major concerns related to p38 inhibitors is high toxicity [13,15]. Therefore, a cytotoxicity assay was performed to determine whether javamide-II-methyl and -ethyl esters with two best scores are cytotoxic to THP-1 cells at the concentrations used in this experiment. At the concentrations between 0–40 μM, the both compounds were considered not cytotoxic (Figure 5).

### 2.7. Effect of Javamide-II-Methyl and -Ethyl Esters on IL-8 and MCP-1 Expression in Differentiated THP-1 Cells

**Figure 6:**
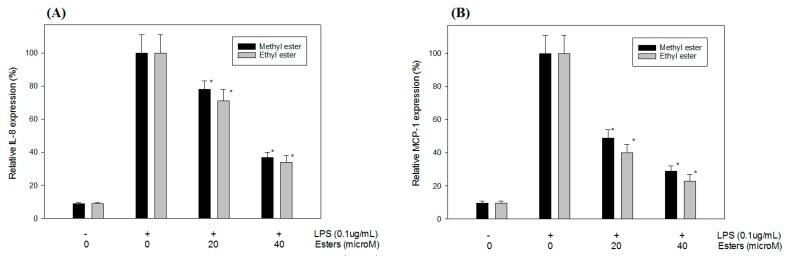
Effects of javamide-II-methyl and -ethyl esters on IL-8 and MCP-1 expression in differentiated THP-1 cells. The samples of IL-8 (**A**) and MCP-1 (**B**) were prepared using differentiated THP-1 cells treated with both esters (0, 20, 40 μM) followed by treatment with lipopolysaccharide (LPS; 0.1 μg/mL) for 18 h as described in “Materials and Methods”. Data points represent the means ± S.D. (*n* = 5). The *p* value was calculated using one-way ANOVA with the Bonferroni's method, and the asterisks (*) indicate significant differences (*p* < 0.05) compared to control.

Because javamide-II-methyl and -ethyl esters were two best score and non-cytotoxic compounds and because IL-8 and MCP-1 productions were reported to be regulated by p38 MAPK [19,20], potential effects of the two esters on IL-8 and MCP-1 expression were investigated as physiological outcomes of p38 inhibition. As expected, the treatment of javamide-II-methyl and -ethyl esters significantly inhibited the production of IL-8 and MCP-1 protein by 22–73% (*p* < 0.05) in the differentiated THP-1 cells (Figure 6). In fact, javamide-II-ethyl ester was slightly better than its methyl ester in suppressing the production of the two cytokines in the differentiated THP-1 cells (Figure 6). These data clearly showed that the javamide analogues with the two best in silico score and in vitro p38 MAP kinase inhibition activities could suppress the production of IL-8 and MCP-1 protein significantly (Figure 6), demonstrating that in silico screening method may be a useful tool in finding candidate inhibitors for p38 MAPK as a first round screening method. In summary, the data from this study suggest that combining in silico and in vitro assay approach may be a useful and effective method in finding new lead compounds for targeted molecules.

## 3. Discussion

Several studies suggest that p38 MAPK can play a significant role in regulating inflammatory cytokines (e.g., IL-1β, IL-8, MCP-1 and TNF-α) and the inhibition of p38 MAPK may be a promising means for suppressing inflammatory cytokines [19,21,22,23]. Therefore, a significant number of p38 inhibitors have been developed and characterized for last ten years. However, only a small number of p38 inhibitors have advanced into clinical phase II studies, due to high toxicity and poor selectivity [13,20]. Therefore, there is still continuing effort to find p38 inhibitors with great efficacy but less toxicity. Recently, virtual screening methods attracted a lot of interest from scientific community, because they provide a rapid and rational approach in finding candidate compounds for targeted molecules. Virtual screening (VS) is to use a high-performance computing method to analyze large databases of potential chemical compounds and to identify possible therapeutic candidates. Particularly, when the molecular structure of the targeted protein is available, the SBD approach is preferable to the LBD approach [9,10]. Therefore, in this paper, potential effects of javamide-I/-II found in coffee were investigated with twenty eight analogues related to p38 MAPK inhibition using in silico modeling program based on SBD approach, because several crystal structures of p38 MAPK are available and because p38 MAPK is relatively well-characterized at the functional, molecular and structural levels. The data from this study clearly showed that javamide analogues demonstrated a range of binding score (Avg. score = −18.1 to −29.3) and some analogues particularly belonging to the group of javamide-II could bind strongly to p38 MAPK. Furthermore, the top three compounds (javamide-II, -methyl and -ethyl ester) to inhibit p38 MAPK most were also found to have high in silico scores. For comparing with p38 inhibitor, the docking data showed that the sum of ΔE value of javamide-II-methyl ester (top score; ΔE = −39.1) was lower than that of the p38 inhibitor SB202190 (ΔE = −28.3), suggesting that javamide-II-methyl ester may bind to the putative binding site better than SB202190. As shown in Figure 2, the conformation of the putative binding site was more accessible to javamide-II-methyl and -ethyl esters in the tested p38 enzyme complex (3HV5) than SB202190, because the esters have a less bulky structure than SB202190. Consequently, the proposed two hydrogen bonds (E71) and (D168) in Figure 2 was not found to be formed with SB202190 due to its structural conformation. Furthermore, the data also suggest that javamide-II analogues (-methyl and -ethyl esters) may inhibit p38 MAPK greater than other analogues. In fact, the javamide-II analogues with the three best in silico scores (Avg. score ≥ −29.1) were found to inhibit p38 MAP kinase stronger (31–33%) than those with less scores (Avg. score < −29.0), demonstrating a positive correlation between the modeling scores and the p38 MAPK inhibition among most of tested javamide analogues, especially the analogues with top three scores. Particularly, javamide-II-*O*-ethyl ester with relatively high in silico score (ΔE = −39.0) could inhibit p38 MAPK (IC_50_ of 9.9 μM) better than the methyl ester with best in silico score (ΔE = −39.1; IC_50_ of 12 μM). Nonetheless, two best-scored analogues (javamide-II-methyl and javamide-II-ethyl) could inhibit p38 MAPK greatly (Table 5) as well as suppress the production of cytokines (IL-8 and MCP-1) significantly as physiological outcomes of the kinase inhibition (Figure 6). Usually, natural compounds often lack efficacy and specificity which are commonly acclaimed by synthetic compounds. However, this study showed that simple esterification of natural compound javamide-II may improve efficacy comparable to synthetic compounds. However, it is known that esters are often labile in the physiological conditions due to the presence of esterase. Therefore, we investigated the stability of javamide-II-methyl and -ethyl esters in tested experimental conditions, but we could not detect the metabolites of the methyl or ethyl esters significantly for 5 h in the conditions, although some increased levels of javamide-II were observed as a metabolite of the methyl and ethyl esters after 24 h. All together, this study clearly demonstrated an outstanding usability of the combining in silico and in vitro assay approach. Especially, this combined approach may be useful in finding bioactive phytochemicals for targeted molecules, because biological activities of many phytochemicals are still unknown.

## 4. Materials and Methods

### 4.1. Materials

Tryptophan, tryptamine, serotonin, cinnamic acid, coumaric acid, caffeic acid, ferulic acid, sinpaic acid, dichloromethane, and other chemicals, were purchased from Sigma Chemical Co. (St. Louis, MO, USA).

### 4.2. Methods

#### 4.2.1. Chemical Synthesis and NMR Analysis

Thirty javamide analogues were synthesized using a method previously described [16,17,18]. Briefly, each phenylpropenoic acid (cinnamic, coumaric, caffeic, ferulic, sinpaic acids) was dissolved in dimethyl sulfoxide (DMSO) and converted to the symmetrical anhydride with 1,3-diisopropylcarbodiimide (DIC). Tryptophan esters were added to the reaction mixture, and the reaction mixture was incubated at room temperatures with a gentle stirring for 12 h. For ester forms, tryptophan was first esterified with corresponding alcohols (methanol, ethanol, propanol) and added to the reaction mixture. The synthesized products were recovered and purified by HPLC (Waters, Milford, MA, USA), as described previously [18]. The chemical structures were verified using HPLC and NMR spectroscopic methods. For NMR experiments, the sample was prepared by dissolving javamide-I (20 mg) in *d*_6_-DMSO (0.75 mL). ^1^H and ^13^C spectra were acquired at ambient temperature on the JEOL BCX-400 NMR spectrometer operating 400 MHz for ^1^H and 100 MHz for ^13^C. Chemical shifts were referenced to DMSO (2.50 ppm for ^1^H, 39.5 ppm for ^13^C).

#### 4.2.2. Molecular Docking

Molecular docking was performed using an algorithm-based docking program ICM-pro (MolSoft, San Diego, CA, USA). The docking program has capability of finding available p38 MAPK protein complexes and storing all necessary information for molecular docking. The pocket analysis of p38 MAP kinase complexes was first conducted for structure optimization, quality assessment and visualization. After that, the built-in 3D database protocol was used to create compound databases for easier screening. The compound database was built based on ICM-pro algorithms, which create compact, indexed compound databases for in silico screening. The generated structure-based model was employed to screen the compound databases using the screen library protocol in ICM-pro. The proposed binding of javamide analogues to the active pocket of each p38 MAPK complexe was determined as the best ranked scoring function, representing the conformational structures with the most favorable binding energy (ΔE). The data of binding energies and score was used to calculate the binding affinity of all docked compounds.

#### 4.2.3. p38 MAPK Assays

Measurements of p38 MAPK activity were performed with the kinase selectivity profiling system CMGC-1 (Promega, Madison, WI, USA). This assay system kit is designed to screen potential inhibitors for p38 MAPK and other kinases in the CMGC Kinase Family. This kit utilizes a non-isotopic, sensitive, and specific method to measure the activities of p38 MAPK. Briefly, kinase working stock, ATP, and substrate working stock solutions were prepared by adding 2.5X kinase buffer to the kinase strips and 100 μM ATP to the substrate strips, respectively. Then, 2 μL kinase solution, 1 μL tested compounds, and 2 μL substrate solution were added to the wells. After that, the reaction was incubated for 60 min and ADP-Glo™ Kinase Assay (Promega, Madison, WI, USA) was performed using a luminometer according to manufacturer’s protocol. The percent activity of p38 MAPK activity was calculated in the presence of inhibitors, inhibition curves were fit to a sigmoidal dose-response (variable slope) equation, and IC_50_ values were calculated using SigmaPlot 11.0 software (Chicago, IL, USA).

#### 4.2.4. Cell Culture

THP-1 cells were purchased from the ATCC (Manassas, VA, USA). Cells were grown in RPMI 1640 medium with l-glutamine containing 10% fetal bovine serum (FBS), 100 units/mL penicillin, and 100 units/mL streptomycin. THP-1 cells were maintained at 37 °C in a humidified atmosphere of 5% CO_2_. THP-1 cells were induced to differentiate into macrophages by incubation with Phorbol-12-Myristate-13-Acetate (PMA; 25 ng/mL) for 48 h. The differentiated cells were treated with analogues followed by LPS treatment (0.1 μg/mL) and incubated for times specified at each experiment.

#### 4.2.5. Determination of IL-8 and MCP-1

IL-8 and MCP-1 levels in the media of the differentiated THP-1 cells treated with javamide-II-methyl and -ethyl esters (0, 20, 40 μM) followed by LPS treatment (0.1 μg/mL) for 18 h were determined using the Human Quantikine IL-8 and MCP-1 Elisa kits from R&D systems (Minneapolis, MN, USA) according to the manufacturer’s protocol.

### 4.3. Statistical Analysis

Treatment effects on the parameters measured were compared by analyzing the means for differences using One-way ANOVA followed by Bonferroni’s test. Differences were considered to be significant when *p* < 0.05. Data points represent the mean ± S.D. of three or more samples.

## Figures and Tables

**Table 1:** p38 Mitogen-activated protein kinase (p38 MAPK) docking scores of javamide-0 and its methyl ester. Docking scores and energy values of p30 MAPK complexes (protein data bank (PDB)) were obtained using a docking program ICM-pro as described in “Materials and Methods”. Average data represents the means ± S.D. (*n* = 14).

p38 (PDB_ID)	Javamide-0		Javamide-0-Methyl	
	Score	ΔE (Kcal/mol)	Score	ΔE (Kcal/mol)
3HV5	−39.2	−47.1	−34.4	−42.3
1W83	−31.7	−39.4	−24.7	−37.1
1W84	−28.4	−37.5	−22.3	−32.1
2PUU	−25.9	−35.4	−21.5	−29.5
3BV2	−27.8	−38.3	−5.9	−12.2
3GC9	−32.6	−39.1	−24.1	−36.3
3GCU	−36.7	−46.6	−24.3	−35.5
3D7Z	−29.7	−38.8	−16.0	−17.0
3DS6	−31.2	−44.2	−21.8	−32.9
3QUD	−18.4	−29.4	−16.0	−27.6
3L8S	−27.2	−39.7	−19.3	−30.2
4R3C	−26.1	−38.2	−17.0	−30.5
4KIP	−27.9	−37.4	−16.0	−25.1
4DLI	−28.7	−36.7	−25.3	−34.5
Average	−20.5 ± 5.1	−24.9 ± 6.2	−18.1 ± 2.7	−24.2 ± 6.7

**Table 2:** Docking scores and energy values of javamide analogues were obtained using a docking program ICM-pro as described in “Materials and Methods”. The asterisks (*) represent the best five inhibition with their ranking.

Groups	Javamide Analogues	Avg. Score	Avg. ΔE
**Javamide-0**	**Javamide-0**	**−20.5 ± 5.1**	**−24.9 ± 6.2**
	**Javamide-0-methyl**	**−18.1 ± 2.7**	**−24.2 ± 6.7**
	**Javamide-0-ethyl**	**−19.4 ± 4.5**	**−21.5 ± 2.3**
	**Javamide-0-propanyl**	**−20.1 ± 2.6**	**−20.8 ± 2.7**
**Javamide-I**	**Javamide-I**	−27.5 ± 2.4 *^5^	**−34.4 ± 6.1**
	**Javamide-I-methyl**	**−27.0 ± 4.3**	**−35.2 ± 2.1**
	**Javamide-I-ethyl**	**−26.1 ± 2.3**	**−30.1 ± 4.8**
	**Javamide-I-propanyl**	**−20.3 ± 2.1**	**−30.4 ± 2.5**
**Javamide-II**	**Javamide-II**	−29.1 ± 7.9 *^3^	**−39.0 ± 7.8**
	**Javamide-II-methyl**	−29.3 ± 4.9 *^1^	**−39.1 ± 4.5**
	**Javamide-II-ethyl**	−29.2 ± 5.8 *^2^	**−39.0 ± 8.7**
	**Javamide-II-propanyl**	**−27.9 ± 8.9**	**−36.9 ± 9.8**
**Javamide-III**	**Javamide-III**	**−26.0 ± 2.7**	**−34.6 ± 7.1**
	**Javamide-III-methyl**	**−22.9 ± 3.4**	**−33.2 ± 8.1**
	**Javamide-III-ethyl**	**−23.6 ± 5.1**	**−30.0 ± 4.6**
	**Javamide-III-propanyl**	**−20.9 ± 7.5**	**−27.0 ± 6.7**
**Javamide-IV**	**Javamide-IV**	**−23.1 ± 2.4**	**−28.4 ± 3.4**
	**Javamide-IV-methyl**	**−20.9 ± 4.5**	**−27.9 ± 5.1**
	**Javamide-IV-ethyl**	**−19.6 ± 2.6**	**−25.0 ± 2.8**
	**Javamide-IV-propanyl**	**−20.1 ± 2.6**	**−25.1 ± 2.7**
**Safflomide**	**Safflomide-0**	**−20.1 ± 2.6**	**−20.8 ± 2.7**
	**Safflomide-I**	**−27.1 ± 4.7**	**−33.8 ± 4.8**
	**Safflomide-II**	−28.3 ± 4.9 *^4^	**−35.2 ± 6.5**
	**Safflomide-III**	**−25.5 ± 4.5**	**−31.5 ± 5.6**
	**Safflomide-IV**	**−22.9 ± 5.1**	**−30.5 ± 9.1**
**Serotomide**	**Serotomide-0**	**−20.1 ± 2.6**	**−20.8 ± 2.7**
	**Serotomide-I**	**−25.6 ± 7.1**	**−30.4 ± 5.8**
	**Serotomide-II**	**−27.4 ± 7.9**	**−33.6 ± 4.1**
	**Serotomide-III**	**−23.9 ± 5.1**	**−31.1 ± 1.6**
	**Serotomide-IV**	**−20.1 ± 3.7**	**−29.6 ± 2.7**

**Table 3:** p38 MAPK docking scores of javamide-I-methyl and SB202190. Docking scores and energy values were obtained using a docking program ICM-pro as described in “Materials and Methods”. Average data represents the means ± S.D. (*n* = 14).

p38 (PDB_ID)	Javamide-II-Methyl Ester		SB202190	
	Score	ΔE (Kcal/mol)	Score	ΔE (Kcal/mol)
3HV5	−39.2	−47.1	−30.1	−40.1
1W83	−31.7	−39.4	−21.2	−35.2
1W84	−28.4	−37.5	−19.6	−28.1
2PUU	−25.9	−35.4	−22.1	−27.1
3BV2	−27.8	−38.3	−10.3	−13.1
3GC9	−32.6	−39.1	−22.3	−33.4
3GCU	−36.7	−46.6	−21.5	−31.8
3D7Z	−29.7	−38.8	−17.1	−19.8
3DS6	−31.2	−44.2	−18.8	−28.4
3QUD	−18.4	−29.4	−17.0	−26.8
3L8S	−27.2	−39.7	−15.6	−27.5
4R3C	−26.1	−38.2	−18.1	−28.5
4KIP	−27.9	−37.4	−17.2	−26.5
4DLI	−28.7	−36.7	−21.4	−31.1
Average	−29.3 ± 4.9 ^(a)^	−39.1 ± 4.5 ^(b)^	−19.4 ± 4.4	−28.3 ± 6.4

^(a)^ *p* < 0.05 javamide-I-methyl vs. SB202190 Score (paired *t*-test); ^(b)^ *p* < 0.05 javamide-I-methyl vs. SB202190 ΔE (paired *t*-test).

**Table 4:** Inhibition of p38 MAP kinase by javamide analogues. The inhibition of p38 by the analogues was determined at the concentrations of 5 μM. Data points are shown as the means ± S.D. (*n* = 4). The asterisks (*) represent the top five inhibition with their ranking.

Concentrations	Javamide Analogues	p38 Inhibition (%)
**Javamide-0**	**Javamide-0**	**−20.5 ± 5.1**
	**Javamide-0-methyl**	**−18.1 ± 2.7**
	**Javamide-0-ethyl**	**−19.4 ± 4.5**
	**Javamide-0-propanyl**	**−20.1 ± 2.6**
**Javamide-I**	**Javamide-I**	**−27.5 ± 2.4**
	**Javamide-I-methyl**	**−27.0 ± 4.3**
	**Javamide-I-ethyl**	**−25.1 ± 2.3**
	**Javamide-I-propanyl**	**−20.3 ± 2.1**
**Javamide-II**	**Javamide-II**	−31.5 ± 7.9 *^3^
	**Javamide-II-methyl**	−31.7 ± 4.9 *^2^
	**Javamide-II-ethyl**	−33.9 ± 5.8 *^1^
	**Javamide-II-propanyl**	**−27.9 ± 8.9**
**Javamide-III**	**Javamide-III**	−28.0 ± 2.7 *^5^
	**Javamide-III-methyl**	**−22.9 ± 3.4**
	**Javamide-III-ethyl**	**−23.6 ± 5.1**
	**Javamide-III-propanyl**	**−20.9 ± 7.5**
**Javamide-IV**	**Javamide-IV**	**−23.1 ± 2.4**
	**Javamide-IV-methyl**	**−20.9 ± 4.5**
	**Javamide-IV-ethyl**	**−19.6 ± 2.6**
	**Javamide-IV-propanyl**	**−20.1 ± 2.6**
**Safflomide**	**Safflomide-0**	**−20.1 ± 2.6**
	**Safflomide-I**	**−27.1 ± 4.7**
	**Safflomide-II**	−28.3 ± 4.9 *^4^
	**Safflomide-III**	**−25.5 ± 4.5**
	**Safflomide-IV**	**−22.9 ± 5.1**
**Serotomide**	**Serotomide-0**	**−20.1 ± 2.6**
	**Serotomide-I**	**−25.6 ± 7.1**
	**Serotomide-II**	**−27.4 ± 7.9**
	**Serotomide-III**	**−23.9 ± 5.1**
	**Serotomide-IV**	**−20.1 ± 3.7**

**Table 5:** p38 inhibition, docking scores and energy values. Docking scores, energy values and p38 inhibition of javamide analogues from both in silico and in vitro experiments were presented with their rankings with the asterisks (*).

Javamide Analogues	p38 Inhibition (%)	Avg. Score	Avg. ΔE
Javamide-II-ethyl	−33.9 ± 5.8 *^1^	−29.2 ± 5.8 *^2^	−39.0 ± 8.7
Javamide-II-methyl	−31.7 ± 4.9 *^2^	−29.3 ± 4.9 *^1^	−39.1 ± 4.5
Javamide-II	−31.5 ± 7.9 *^3^	−29.1 ± 7.9 *^3^	−39.0 ± 7.8
Safflomide-II	−28.3 ± 4.9 *^4^	−28.3 ± 4.9 *^4^	−35.2 ± 6.5
Javamide-III	−28.0 ± 2.7 *^5^	−26.0 ± 2.7	−34.6 ± 7.1
Javamide-I	−27.5 ± 2.4	−27.5 ± 2.4 *^5^	−34.4 ± 6.1
